# Infrastructure-Less Communication Platform for Off-The-Shelf Android Smartphones

**DOI:** 10.3390/s18030776

**Published:** 2018-03-04

**Authors:** Takuma Oide, Toru Abe, Takuo Suganuma

**Affiliations:** 1 Graduate School of Information Sciences, Tohoku University, Miyagi 980-8577, Japan; 2 Cyberscience Center Tohoku University, Miyagi 980-8577, Japan; beto@tohoku.ac.jp (T.A.); suganuma@tohoku.ac.jp (T.S.)

**Keywords:** device-to-device, multi-hop communication, Wi-Fi direct, smartphone, android

## Abstract

As smartphones and other small portable devices become more sophisticated and popular, opportunities for communication and information sharing among such device users have increased. In particular, since it is known that infrastructure-less device-to-device (D2D) communication platforms consisting only of such devices are excellent in terms of, for example, bandwidth efficiency, efforts are being made to merge their information sharing capabilities with conventional infrastructure. However, efficient multi-hop communication is difficult with the D2D communication protocol, and many conventional D2D communication platforms require modifications of the protocol and terminal operating systems (OSs). In response to these issues, this paper reports on a proposed tree-structured D2D communication platform for Android devices that combines Wi-Fi Direct and Wi-Fi functions. The proposed platform, which is expected to be used with general Android 4.0 (or higher) OS equipped terminals, makes it possible to construct an ad hoc network instantaneously without sharing prior knowledge among participating devices. We will show the feasibility of our proposed platform through its design and demonstrate the implementation of a prototype using real devices. In addition, we will report on our investigation into communication delays and stability based on the number of hops and on terminal performance through experimental confirmation experiments.

## 1. Introduction

As smartphones and other small portable devices become more sophisticated and popular, sensor data collected from these devices are being utilized at a rapidly expanding rate [1,2,3]. In particular, recent research on service models in which users actively or passively act as data providers or relayers based on the people-centric networking paradigm [4,5], which includes users and their devices in the system components, has been conducted for the purpose of effectively utilizing user-owned device resources. With this in mind, we are now conducting advanced research and development into a sensor-based application building model known as a contract-oriented sensor-based application platform (COSAP), which can flexibly control data disclosure settings based on the concept of “contracts” between data consumers and providers [6]. A COSAP is an infrastructure-less data distribution platform in which sensor data can be freely distributed by participating in peer-to-peer (P2P) networks built between users. However, studies on methods for constructing communication platforms that connect user devices, which are necessary for configuring P2P networks, have not advanced significantly to date.

On the other hand, expectations for device-to-device (D2D) communication platforms that can be utilized as communication bases for connecting portable devices to each other are now increasing [7]. D2D communication platforms, which are infrastructure-less communication platforms that use the resources of terminals owned by users, have been shown to be superior to conventional infrastructure in terms of bandwidth efficiency, power efficiency, communication speed, etc. [7]. Since D2D communication makes it possible to build networks and share data without requiring special components, it is expected to be especially useful in the event of a disaster [8,9]. Moreover, since the penetration rate of Android terminals is more than 50% in many countries [10], with the penetration rate of devices equipped with the Android 4.0 operating system (OS) at more than 99.6% as of January 2018 [11], this research aims specifically at realizing a D2D communication platform for Android devices.

Widely spreading D2D protocols include Wi-Fi Direct and Bluetooth. In addition, in mobile network, LTE/LTE-A introduces the concept of D2D communication, which is expected as an effective offloading strategy [12,13]. Among these, we use Wi-Fi Direct [14] to create our D2D communication platform. Wi-Fi Direct is a protocol installed in Android 4.0 or higher devices that it is superior to protocols such as Bluetooth in terms of communication speed and effective range of radio waves [15,16]. By applying Wi-Fi Direct, it becomes possible to freely establish a local network using only smartphones without requiring special devices such as base station. However, Wi-Fi Direct assumes single-hop communication within a group between one group owner (GO) and multiple clients (CLs) associated with that GO, and does not support multi-hop communication among multiple groups by itself. Additionally, the Internet Protocol (IP) addresses of all GOs are fixed at 192.168.49.1/24, and each CL is assigned a random IP address by the GO using the Dynamic Host Communication Protocol (DHCP). Because of this, it is possible that IP address conflict will occur between devices during communication between groups, and routing using IP addresses is difficult. There are some previous works on multi-hop D2D network with Wi-Fi Direct [17,18,19,20,21,22], however, they have limitations when applying to off-the-shelf smartphones in practical use cases. For example, some of them need *rooted* devices [17] and/or prior setting sharing with peripheral devices [18,19], which greatly reduce the scope of application.

Therefore, in this research, we propose a tree-structured D2D communication platform in which GO controls are distributed topologically among the participating devices. On this platform, by using Wi-Fi Direct and Wi-Fi in combination, we can establish connective relationships between the groups and build a tree-type topology. In addition, GO manages the topology of each group in a decentralized manner, and by sharing this topology information among GOs, achieves routing based on the topology relationship between devices.

The contributions of this paper are as follows:

- We introduce a tree-structured network topology construction method which can connect multiple Wi-Fi Direct groups without sharing prior knowledge. Since the method is assumed to be performed autonomously and decentralized, users can construct an ad hoc private D2D network.
- We also introduce a novel routing method based on the relationship of the tree topology rather than IP address. Since arbitrary device ID is used for device identification, there is no need to worry about IP address conflicts.
- We show that the above functions can be realized on off-the-shelf smartphones. Our experiments indicate that users can freely communicate with unknown peripheral smartphones with low delay even when no local network or hotspot is available.

The remainder of this paper is organized as follows: In Section 2, we will describe Wi-Fi Direct, which is the D2D protocol adopted in this research and related research, and describe their characteristics and problems. Next, to clarify the Wi-Fi Direct specifications, we will report on the results of our preliminary experiments in Section 3 and explain our design for the proposed platform based on those obtained results in Section 4. After that, we will implement the proposed platform on real devices and consider the feasibility of the platform via the evaluation experiment discussed in Section 5. Finally, in Section 6, we conclude this paper and discuss future issues.

## 2. Related Works

### 2.1. Wi-Fi Direct

Wi-Fi Direct [14] is a communication protocol formulated by the Wi-Fi Alliance, which has been installed as a standard from the Android 4.0 OS. It is superior to the Bluetooth protocol in terms of communication speed and effective range of radio waves [15,16,20]. However, since Wi-Fi Direct only supports intra-group single-hop communication between one GO and its clients (CLs), only a star-type topology can be constructed. Nevertheless, since the network interface controller (NIC) used in Wi-Fi Direct is a dedicated logical P2P-side controller rather than the typical Wi-Fi-side controller used for normal Wi-Fi communications, Wi-Fi and Wi-Fi Direct can be used together by properly interfacing these protocols. In addition, since a GO is recognized as an access point (AP) for the surrounding devices in normal Wi-Fi, a Wi-Fi connection to a GO is possible even for devices that are not equipped with Wi-Fi Direct. The IP address assigned to the P2P-side NIC of the GO is fixed at 192.168.49.1/24, while the IP address of the P2P-side NIC (or the Wi-Fi-side NIC) of the CL connected to the GO is randomly assigned by the GO via the DHCP.

Wi-Fi Direct supports three forms of group construction [23].

1. Standard: After discovering a neighboring device, negotiations are conducted to determine which device will be the GO; decide the roles of the other devices; and assign IP addresses, etc.
2. Autonomous: If either device is already a GO, then it assumes that role without negotiation and proceeds to assign IP addresses, etc.
3. Persistent: If devices have previously experienced connection, then the previous GO resumes that role without renegotiating and proceeds to assign IP addresses, etc.

As shown above, the process of group construction is roughly divided into Discovery phase, Negotiation phase, and Address configuration phase. In the Discovery phase, devices periodically send/scan Probe Requests using channels 1, 6, or 11 in the 2.4 GHz band. Device names and status (GO  or not) are included in the frame. In the Negotiation phase, an intent value (IV) is exchanged between the devices and the one that has the highest value becomes the GO. The calculation of the IV depends on the implementation. Finally, in the Address configuration phase, the GO assigns IP addresses of its CLs via DHCP. Additionally, Wi-Fi Direct provides advertising of Local Service, namely Wi-Fi P2P Service Discovery. This is originally used to communicate between applications even when no local network is available.

Note that using actual devices, it has been confirmed that the Autonomous mode completes in about half the time required for the other forms [8,23].

### 2.2. Wi-Fi Direct-Based Multi-Hop Communication Platform

Since Wi-Fi Direct places a heavier burden on the GO than on a CL, it is important to properly select the device that will become the GO. Additionally, when targeting only single-hop communication, GO  selection is especially important because the number of CLs that can be connected is determined by the physical position of the GO. For that reason, numerous studies have been conducted [24,25,26,27,28,29] on determining the appropriate GO selection method in order to improve the performance of Wi-Fi Direct. However, since Wi-Fi Direct was originally designed for inter-group single-hop communication, using it efficiently to perform multi-hop D2D communication is difficult.

For this reason, a number of studies aimed at devising methods for constructing D2D communication platforms capable of multi-hop communication for smartphones have been conducted. In  [8,15], methods of performing multi-hop communication using only Wi-Fi Direct were proposed. However, in these methods, each device is constantly kept waiting as a GO, and a device only becomes a CL when it needs to connect to the GO of a transfer destination to forward a message, thereby realizing multi-hop communication. Unfortunately, since it is necessary to change roles and rebuild the group after every transfer, the methods consume significant amounts of time [23] and simultaneous transmission of multiple messages can result in collisions that in turn result in failures. A method in  [25] also uses Wi-Fi Direct only; however, since each group is independent, the reachability of messages to nodes in other groups is uncertain. In  [9], a method combining Wi-Fi Direct and Bluetooth was proposed. However, in that method, Bluetooth is also used for data transfer, and it has been shown in the literature that not only will the transfer rate be slow but also crosstalk with Wi-Fi will occur.

In recent years, based on the Wi-Fi Direct characteristics mentioned in the previous section, methods that can be used to construct D2D communication platforms have been studied by using different logical interfaces separately for Wi-Fi Direct and Wi-Fi [17,18,19,20,21,22]. In  [17], inter-group communication is realized by modifying the source code of the Android OS in order to make each GO have a different subnet. Additionally, since it was indicated in  [18,19] that a device having a specific connection relationship with a plurality of GOs can arbitrarily communicate with each GO, inter-group communication methods based on the assumption of such “relay devices” were proposed [20,21]. Furthermore, a technique for realizing the same function by effectively using multicast communication is shown in [22].

### 2.3. Challenges

In studies targeting Wi-Fi Direct, there are numerous cases where the mobile device OSs have been rooted and modified for the purpose of improving stability and performance. However, it is difficult to apply these approaches to cases involving information sharing in emergencies [8] and the sharing of sensor data using user devices in the Internet of Things (IoT) environment. Furthermore, in research on multi-hop communication platforms, there are limitations such as the occurrence of numerous delays at the time of transfer and the necessity of setting up sharing between devices before connection.

In this research, we will realize a D2D communication platform that simultaneously achieves the following to solve the indicated problem.

**(R1)**  Connection with unknown devices without prior setting sharing: It is difficult to build a topology between the devices without modifying their OS and/or sharing settings with other devices, such as reporting their IP addresses.
**(R2)**  Low-latency multi-hop communication among arbitrary devices: Since Wi-Fi Direct provides only single-hop communication within the star topology, it is difficult to construct efficient low-delay communication with arbitrary devices.

In addition, as a feature of our research compared with related research satisfying the above-mentioned requirement [20], dynamic adaptation to the environment can be cited. In this research, to achieve compatibility with the IoT environment, our design and implementation assumes an environment where unknown devices move dynamically. In contrast, in the method of  [20], it is necessary to synchronize peripheral devices several times to determine the optimum topology configuration, which means that the network may be vulnerable to sudden participation and withdrawal of devices. Additionally, the method evaluation was performed in a very close environment where no devices were shielded. At the time of actual application, the risk of topology construction failure may be increased due to packet loss caused by imposed shielding or by increased distance between devices. Therefore, in this research, we will prioritize the establishment and maintenance of our communication platform under dynamic environments, and will not consider the appropriateness of the topology to be constructed.

## 3. Preliminary Experiment

### 3.1. Setup

Because Wi-Fi Direct and Wi-Fi share one hardware as two logical NICs, their priorities and reference order depend on OS implementation. Therefore, before designing our platform which uses both NICs, it is necessary to clarify its characteristics by experiments using real devices. In particular, since the address space of IP address assigned to both NICs is the same (192.168.49.0/24), communication between devices fails in some cases such as in the following case.

- When sending a message to the GO of a group to which it belongs as a CL, the destination IP address is equal to the IP address assigned to its own P2P-side NIC (192.168.49.1/24), which means that it is processed as a message addressed to itself.

Therefore, in this preliminary experiment, the possibility of transmission and reception between each device within a single Wi-Fi Direct group is verified for each device role and the usage situations of both NICs.

For our preliminary experiment, we first installed an application to three ZenFone 3 (Android 7.0) devices. After that, we launched one arbitrary device as the GO, invited in two other devices, and built a Wi-Fi Direct group. Then, we attempted to send messages between the devices by unicast communication and by broadcast communication to determine whether reception was successful.

Considering the role (GO/CL) and NIC usage situation (only on the P2P-side/only on the Wi-Fi-side/both), we selected twelve patterns that were able to verify all combinations.

### 3.2. Results

**Figure 1:**
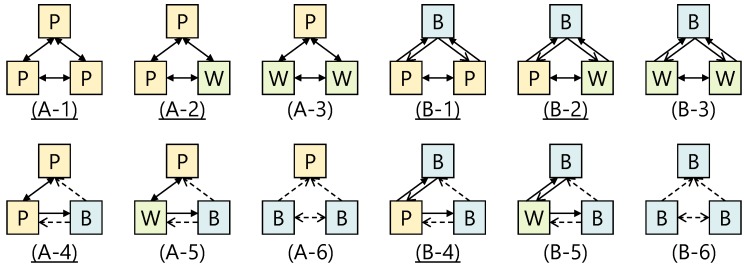
Results of preliminary experiment. The top upper box of each topology is the GO and the two lower boxes are CLs. The solid arrows represent unicast communication is available, the dotted arrows represent broadcast communication is available, and the filled solid arrows represent both communications are available.

Figure 1 shows the results of summarizing whether communication is possible in each topology. Note that P indicates a device using only the P2P-side NIC, whereas W indicates a device using only the Wi-Fi-side NIC, and B indicates a device using both NICs. Since a CL can connect to the GO through both NICs, all P, W, and B situations are conceivable. However, since a GO always uses the P2P-side NIC, we do not think a situation will arise in which the GO is W.

The results of our preliminary experiment confirmed that bidirectional communication is possible in both unicast and broadcast communication on the paths between P-P, P-W, and W-W using only one-sided logical NIC. On the other hand, in communication with the B which is using both NICs, it was found that availability depends not only on the NIC usage status but also on the role of each device.

Based on the above results, we consider the topology used in the proposed platform. First, we exclude topologies (A-6 and B-6) where bidirectional communication is not possible between the GO and the CL. This is due to the collision of IP addresses between devices as described above. This problem can be solved by rewriting the Android OS, changing the reference order and priority of the routing table. However, we do not adopt this solution because rewriting OS significantly narrows the scope of application of the platform. In addition, although the NIC of the CL connecting to the GO can be controlled by the GO, under a Wi-Fi connection, it is impossible to suppress situations in which the CL becomes a GO of a new group (W→B). Because of this, we will also exclude the four topologies (A-3, A-5, B-3, and B-5) that can transition to the above-mentioned topology where bidirectional communication is impossible. After taking the above exclusions into consideration, our proposed platform adopts the six topologies with names underlined in Figure 1.

## 4. Tree-Type Structured D2D Communication Platform

In this research, we propose a structured D2D infrastructure-less communication platform by off-the-shelf smartphones (which is hereafter referred to as ICOS) for Android devices. Focusing on features related to the fact that Wi-Fi Direct and Wi-Fi use different logical NICs described in Section 2.1, ICOS uses the P2P-side NIC for intra-group connection and the Wi-Fi-side NIC for inter-group connection. The result is a tree-type topology that does not require reconstruction after every message transfer. In addition, by introducing a relay node (RN) to provide a new function alongside the GO and CL roles, multi-hop communication between groups can be realized.

### 4.1. Topology Construction Mechanism

This mechanism is used to establish connectivity with unknown peripheral devices in ad hoc environments by using Wi-Fi and Wi-Fi Direct in combination. At this time, each GO autonomously controls the connection interface with the CLs in the group so that it is structured as a tree-type topology with a certain GO as the root. Control by GO can be performed using only the information exchanged by the original Wi-Fi Direct settings, which means that users do not need to input setting information or information on other devices into their devices in advance. With this feature, a tree-type topology capable of multi-hop communication is established without the need for prior setting, and achieves (R1).

Here, an RN is newly introduced as a device role, and one or more RNs are elected from CLs of each group. Note that connection with GO using only the P2P-side NIC is permitted. This corresponds to the P that always exists on CL side in the six adopted topologies selected based on the considerations discussed in Section 3, and plays in relaying messages from the GO to other CLs in the (A-4) and (B-4) topologies. For a CL, there is no limitation on the NIC connection.

The topology is constructed by the following procedure.

1. A user who wishes to construct a D2D network makes its device a GO and invites peripheral devices to its group as CLs.
2. The role of RN is given to the first connected CL and only P2P connection is allowed.
3. The information necessary for Wi-Fi connection is given to CLs that connect after that, and any connection is allowed.
4. A CL that has switched to a Wi-Fi connection becomes the GO of a new group and waits for connections from other devices.
5. The GO periodically advertises its group information to all devices connected to itself.

**Figure 2:**
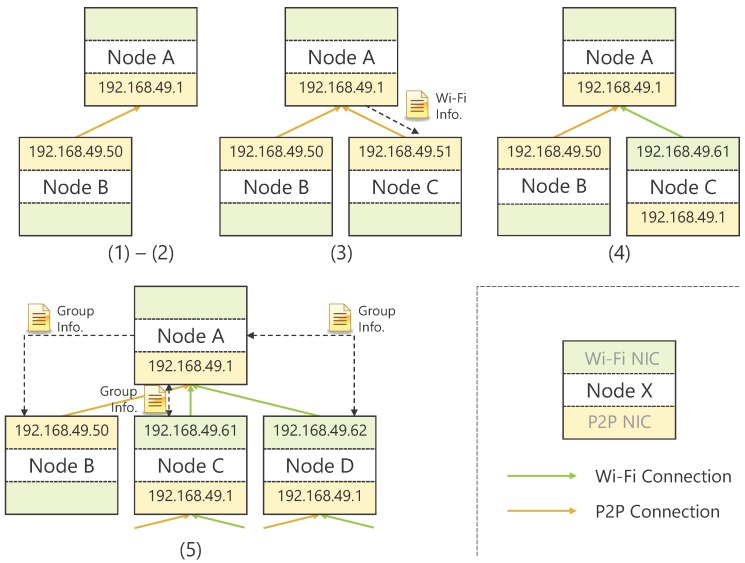
Steps for topology construction. Each number corresponds to the processing procedure in the text.

Figure 2 shows the topology construction procedure. In Steps (1) and (2), both devices establish a connection using the Wi-Fi Direct function. At this time, by setting one of them as the GO in advance, the time required for group construction is shortened. Additionally, in Step (3), when there is a connection of the second and subsequent CLs, GO provides its AP information (service set identifiers (SSIDs) and Passphrases) necessary for Wi-Fi connection. Based on this information, CLs decide whether to continue the P2P connection or switch to a Wi-Fi connection, and the device that has switched to a Wi-Fi connection becomes the GO of a new group and waits for connections from other devices in Step (4). Thereafter, in the step (5) the GO periodically exchanges its group information (GO  information, RN information, CL list, and routing table) with the surrounding devices and always shares the latest topology information. However, the periodical exchange of information in this step is not essential. Since GO can detect an event that changes in CLs being connected to the GO by the function of Wi-Fi Direct, thus it will suffice to share information at that time. The periodical information exchange is useful for obtaining the latest information on devices not directly connected, but it is a trade-off with increase in network traffic.

**Figure 3:**
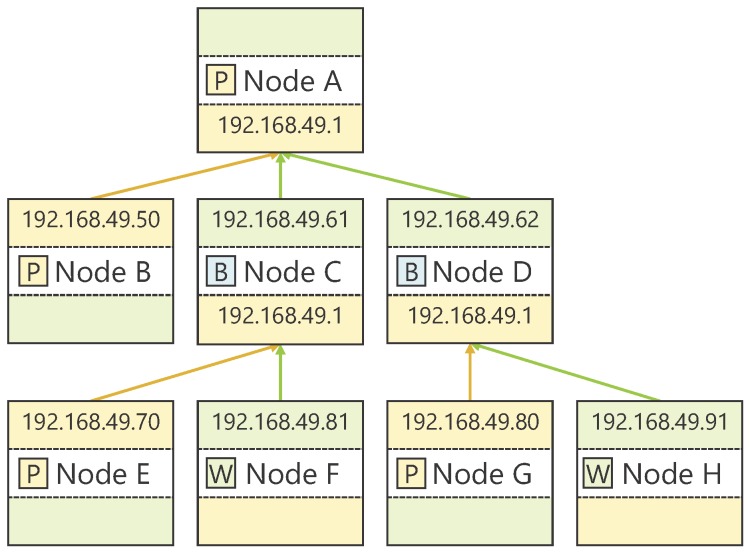
Example of topology construction. The IP address assigned to the P2P-side NIC of GO is fixed at 192.168.49.1/24, while the other IP addresses are randomly assigned.

Figure 3 shows a topology construction example. At this time, Nodes A, C, and D are GOs, and Nodes B, E, and G are the RNs of each group, respectively. From that point, Nodes A, C, and D accept connections from new devices, or Nodes F and H become new GOs, thereby expanding the topology.

**Figure 4:**
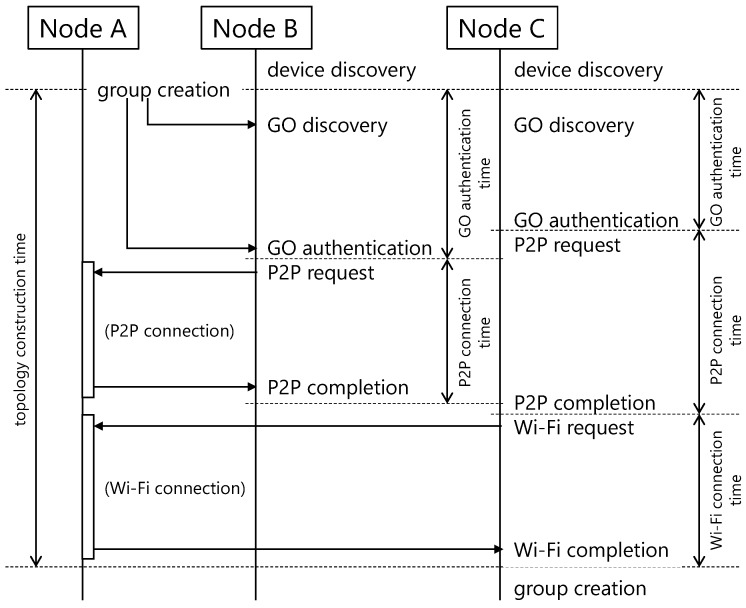
Sequence diagram of topology construction mechanism.

Figure 4 shows the detailed diagram of the topology construction mechanism. The behaviors of each device in the figure are listed below.

1. Each node transitions to the “device discovery” state using ICOS in order to discover peripheral devices.
2. Node A executes “group creation” as the GO and starts constructing a D2D network.
3. Node B and Node C discover Node A (“GO discovery”) and authenticate that it is a GO that is using ICOS (“GO authentication”).
4. Node B and Node C attempt a P2P connection to Node A using Wi-Fi Direct (“P2P request”).
5. Node A establishes P2P connections with the requesting devices (“P2P completion”).
6. Node A sends the Wi-Fi connection information that was established during the second connection to Node C.
7. Node C disconnects the P2P connection to Node A and attempts a Wi-Fi connection (“Wi-Fi request”).
8. Node A establishes a Wi-Fi connection with the requesting device (“Wi-Fi completion”).
9. Node C becomes the new group’s GO and waits for connection requests from other devices (“group creation”).

The specific processing performed in each state is as follows. However, exchanging messages using Bluetooth is performed only on Android 5.0 or higher devices.

**device** **discovery** starts searching neighboring devices and Local Services in Wi-Fi Direct and begins scanning Advertise message in Bluetooth. Searching for devices and services is accomplished with standard functions of Wi-Fi Direct. Advertise message, including device name and status (GO or not), is distributed by advertise/scan functions of Bluetooth. Note that since the search function in Wi-Fi Direct is particularly unstable, the system will retry the search every 10 s. However, the retry is not performed during either the P2P or Wi-Fi connection requests.
**group** **creation** creates a new group as the GO, registers Local Services in Wi-Fi Direct, and then sends an Advertise message in Bluetooth. At this time, by including the ICOS-specific character string in the Local Service service name and the Advertise message payload, the new GO advertises that it is a device currently using ICOS to connect with peripheral devices.
**GO** **discovery** obtains device information in Wi-Fi Direct. At this time, only the GO devices identified from the acquired device information are held. At this point, it is not possible to determine whether the discovered devices are currently using ICOS.
**GO** **authentication** obtains Local Service information in Wi-Fi Direct or the Advertise message in Bluetooth. At this time, it determines whether ICOS-specific character string is included in the service name of the Local Service or the Advertise message payload, and only retains information on devices having information satisfying that requirement. Next, by comparing it with the information obtained by “GO discovery”, it authenticates that it is a GO that is using ICOS.
**P2P** **request** requests a connection to the discovered GO in Wi-Fi Direct. However, since the Wi-Fi Direct connection request may fail, the system retries the connection request every 10 to 20 s. The time between reconnection requests is determined based on the Wi-Fi Direct state of the device requesting the connection. More specifically, when the state is WifiP2pDevice.INVITED, a retry is executed 10 s after the first request is sent, but if it is WifiP2pDevice.CONNECTED, then the waiting time is extended by 10 s.
**P2P** **completion** holds group information sent from the GO. At this time, if the device is registered in the group information as a CL instead of an RN, it may continue to “Wi-Fi request”.
**Wi-Fi** **request** leaves the group in Wi-Fi Direct and attempts to connect to the GO by Wi-Fi. Since the Wi-Fi connection request may fail in the same way as the “P2P request”, the system retries the connection request every 10 s.
**Wi-Fi** **completion** immediately transits to the “group creation” state.

### 4.2. Multi-Hop Communication Mechanism

This mechanism constructs a routing table based on the relationship with the other devices in the structured topology and transfers the message. At this time, since each GO holds the topology information shared when the topology construction, connectivity with an arbitrary device is established without requiring user input and setting pre-sharing. In addition, it is possible to send messages to other devices by unicast, and it is unnecessary to reconstruct the topology accompanying the message transfers. This feature realizes flexible routing according to circumstances and uses, and achieves (R2).

As already mentioned, it is not possible to communicate using the IP address due to multiple Wi-Fi Direct groups connected. Therefore, the platform uses device ID-based routing overlaid on IP address-based routing. Devices can use any information (phone number, MAC address, etc.) that can uniquely identify a device, as its own device ID. IP address is only used for single-hop communication with directly connected devices.

Considering the connectivity between devices in the topologies selected in Section 3, each device updates its routing table in the following procedure based on its own role.

GO When a message arrives from devices of its group, the GO updates both the group information and routing table to ensure that group members can always communicate with each other via the RN. When messages arrive from other groups, only their information is updated.
CL When a message arrives from devices of its group, the CL updates the information about both the group and the routing table to ensure that they can always communicate with each other directly. When messages arrive from other groups, only their information is updated.
RN Same as CL.

Additionally, the message transmission form is determined based on the following rules.

$$\left\{\begin{array}{cc} \mathrm{Unicast}\;\mathrm{communication} & \left(\mathrm{GO}\to \mathrm{RN}\right)\\ \mathrm{Broadcast}\;\mathrm{communication} & \left(\mathrm{CL},\mathrm{RN}\to \mathrm{GO},\mathrm{CL},\mathrm{RN}\right) \end{array}\right.$$

The reason the GO always forwards messages via its RN is because there is no means to communicate from the GO side when a CL is B (see (A-4) and (B-4) in Figure 1). Since the only valid message transfer model at this time is unicast, a unicast communication to the RN is always adopted for communication from the GO. On the other hand, since direct broadcast communication can be performed to any neighbor device from CLs and the RN, they always use direct broadcast communication. Although all neighboring devices will receive the same message when broadcast communication is used, the destination device ID is confirmed during implementation by referring to the independently set message header and any messages that are not headed with their device ID are discarded.

**Figure 5:**
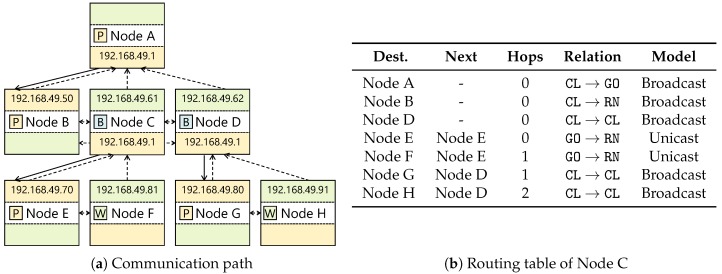
Routing table example: (**a**) The solid lines stand for unicast communication, and the dotted lines stand for broadcast communication; and (**b**) routing table of Node C.

Figure 5 shows an example of routing table construction in Figure 3. As shown in Figure 5a, the communication model between devices is only unicast communication to the RN at the time of transmission from the GO to the device in its group, and all other communications between group members are direct broadcasts.

The routing table of Node C at this time is shown in Figure 5b. For communication to Nodes A, B, and D, direct communication by broadcast is applied since they belong to the same group that Node C belongs to as a CL. Additionally, as for communication to Nodes E and F, because they belong to the same group that Node C belongs as a GO, the route via Node E, which is the RN, is applied by unicast. Furthermore, even though there is no direct connection relationship with Nodes G and H, since its route information is obtainable when the routing table is shared with Node D, that node is registered as the next hop destination. It is to be noted that the number of hops is aimed at preventing duplication of routes generated when exchanging routing tables with neighboring devices, and the route having the minimum hop count is always adopted. Since the shortest route is uniquely determined from the topology structure, the routing table held by each device always converges after a certain period of time elapses unless devices move. Note that relationship between devices and communication model are just referred to at the time of message transmission, and are not included in the routing table information shared with others.

**Figure 6:**
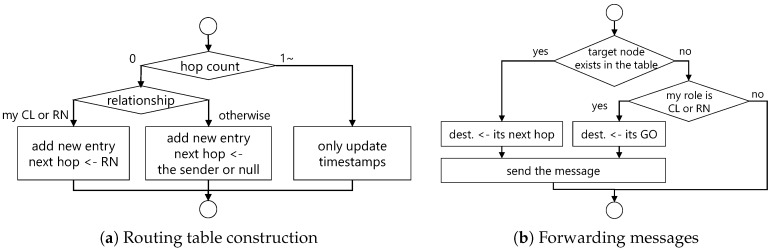
Flowchart of multi-hop communication mechanism.

Figure 6 shows flowcharts of routing table construction and message transfer. During routing table construction, each time a device receives a message, it executes the flow shown in Figure 6a. More specifically, when receiving a message directly, the information for both the sender and devices included in the payload is added as a new entry. At this time, if the device is a GO and the sender is a CL or an RN, then the next hop is overwritten as an RN. Otherwise, next hop is overwritten as the sender or a null (direct communication). Additionally, when the message is received via one or more relays, new entries are not added and the only the timestamp of corresponding entries is updated. The timestamp is used to determine whether a device has left the network for reasons such as movement. In this implementation, HELLO messages are periodically sent to entries that do not update for more than 10 s in order to confirm their existence, and entries that have not been updated for more than 60 s are deemed to have left the network and are deleted from the routing table.

In message transfers, when each device transmits a message, it executes the flow shown in Figure 6b and determines the next hop device. More specifically, when the destination device information exists in its routing table, the next hop described in the entry is designated as the forwarding destination. Additionally, even if the destination device information does not exist in its routing table, if the device itself participates in ICOS network as a CL or an RN, it designates the GO as the forwarding destination and transmits the message. In other cases, no message is sent.

## 5. Evaluation

### 5.1. Implementation of ICOS-Based Application

For the evaluation, as an ICOS application example, we report on the implementation of a sensor data sharing system that can distribute sensor data among nearby devices. In this system, the topology construction mechanism described in Section 4.1 automatically detects peripheral devices that are running ICOS, and then constructs a private ad hoc D2D network consisting solely of those devices. After that, the multi-hop communication mechanism described in Section 4.2 acquires and displays specific sensor data from arbitrary devices participating in the network instantly. By implementing the application that visualizes sharing of sensor data, it becomes possible to intuitively understand the connectivity between devices in the subsequent evaluation experiments.

Table 1 shows the list of Android devices used for the implementation and operation verification of this system. The implementation environment is shown below.

- **IDE**: Android Studio 3.0.1
- **Compiled SDK**: Version 25 (Android 7.1)
- **minimum SDK**: Version 18 (Android 4.3)

**Figure 7:**
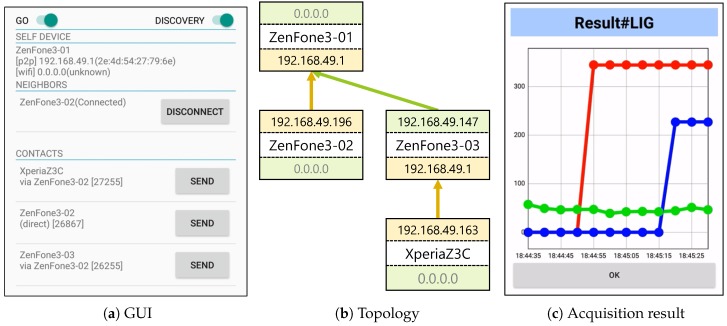
Example of running system.

Figure 7 shows the implemented sensor data sharing system. Figure 7a is the graphical user interface (GUI) of ZenFone3-01 in the topology configuration shown in Figure 7b. In the GUI display, the device’s own information is shown in the upper row, information on neighboring devices in Wi-Fi Direct is shown in the middle row, and information on devices participating in the same ICOS network is shown in the lower row. From this figure, it can be confirmed that the usage status of NICs of its device is reflected correctly and the routing table is correctly shared. Furthermore, Figure 7c shows a state in which illuminance sensor values are acquired from peripheral devices. As can be seen in the figure, instant sensor data acquisition is performed for each peripheral device from the point in time at which it became possible to communicate with its affiliated devices.

This system is composed of the ICOS functions and the sensor data sharing mechanism. When providing sensor data, owners decide whether to provide data for each built-in device sensor. Thereafter, the system assigns a specific tag to the end of the device’s name based on the type of sensor data sharing permitted by the owners, and thereafter transmits messages with the device name. As a result, the routing tables of other devices include not only the device name but also the types of sensor data that its owner allows to be shared. This implementation has five built-in sensor types: a position sensor, an atmospheric pressure sensor, an illuminance sensor, an air temperature sensor, and a humidity sensor. Furthermore, when acquiring sensor data, owners search for devices by including a tag for a specific sensor type from their routing table, and also send a sensor data request message to the target devices at predetermined time intervals. Thereafter, the acquired sensor data are visualized and displayed. Note that all CLs choose to switch to Wi-Fi connection regardless of its situation in this implementation.

### 5.2. Operation Verification of Sensor Data Sharing System

#### 5.2.1. Setup

This experiment was conducted to verify that the system achieves instant communication with arbitrary peripheral devices by constructing an ad hoc D2D network using the ICOS function without requiring pre-setting sharing with other devices. We set up four scenarios to verify this achievement by observing the acquisition of sensor data executed in the system. The devices used in this experiment are Devices 1–4 in Table 1, and no preliminary setting adjustments were made other than installing the ICOS system in advance. Furthermore, as common conditions in each scenario, the illuminance sensor is registered in advance both by Devices 1–3 (as providers) and by Device 4 (as a consumer). In addition, the distance between devices within the same room was set at 10 cm to reduce the possibility of packet loss due to the communication environment. A qualitative evaluation of the topology construction mechanism and the multi-hop communication mechanism will be performed in later experiments. Note that all devices launched only ICOS, and enabled only Wi-Fi, Bluetooth, and GPS. However, Device 4 was connected to the mobile network.

**Figure 8:**
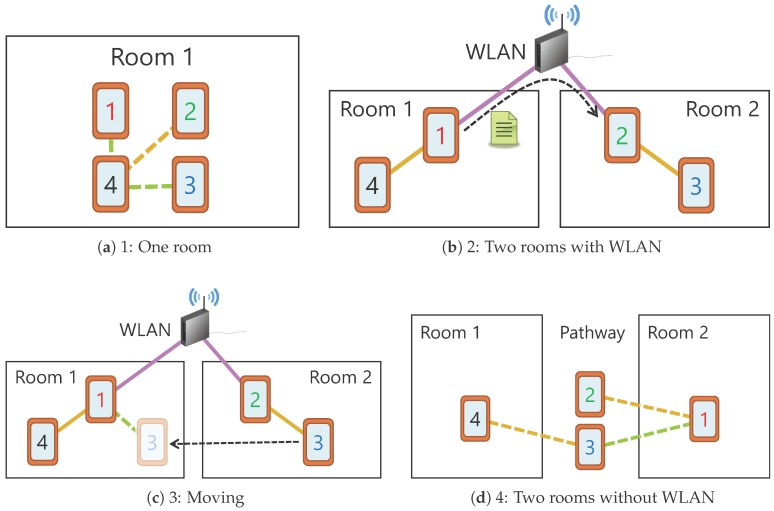
Operation verification scenarios. Finding devices in the remote room was impossible by Wi-Fi Direct or Bluetooth, but devices in the pathway could discover devices in each room.

Figure 8 shows outline of the scenarios carried out in this experiment. The yellow lines represent the P2P connection, the green lines represent the Wi-Fi connection, and the purple lines represent the wireless local area network (WLAN) connection. In addition, the solid lines show the connections established before the experiment starts and the dotted lines show the connections established after the experiment starts.

In Scenario 1, after placing all devices in the same room and putting them in “create group” state, Device 4 executes “create group”, thereby starting sensor data sharing among the connected devices. Through this scenario, we will confirm whether peripheral devices can autonomously construct a private D2D network and start sensor data sharing. In Scenario 2, Devices 1 and 4, and Devices 2 and 3 are placed in the same rooms and D2D networks are constructed in advance, as in Scenario 1. In addition, Devices 1 and 2 are connected to a common WLAN in advance. Thereafter, Device 1 sends a HELLO message to Device 2 in order to establish connectivity between the two rooms via the WLAN. Through this scenario, we will confirm whether Device 4 establishes a connection with the remote room and starts acquiring sensor data. In Scenario 3, similarly to Scenario 2, devices in the two rooms are connected by the WLAN and sensor data are being shared among all devices. Thereafter, Device 3 moves to the remote room without any prior notification. Through this scenario, we will confirm whether Device 3 reconnects to the network at the destination room and restarts sensor data sharing. Finally, in Scenario 4, devices are arranged in two different rooms with a pathway that acts as a relay point, and Device 1 starts sensor data sharing after “create group” execution. Through this scenario, we will confirm whether connections between devices in two rooms are possible without using infrastructure such as a WLAN, and whether sensor data are shared among them.

#### 5.2.2. Results

**Figure 9:**
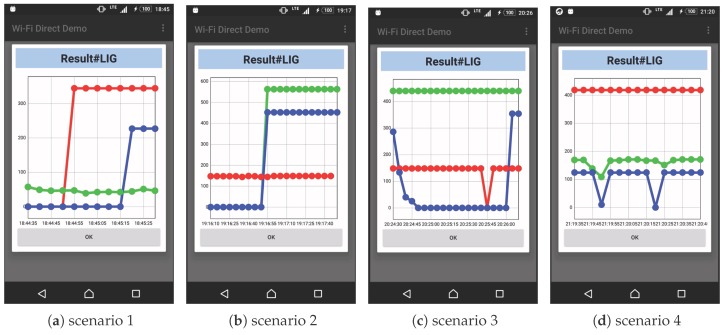
Operation verification results. The X-axis represents the time when messages arrived, and the Y-axis represents the acquired illuminance sensor value (lux). The color of the graph corresponds to the character color of the device number in Figure 8.

Figure 9 shows the experimental results. The graphs in the figure shows how Device 4 acquires sensor data from other devices. In this setup, sensor data are distributed as long as the connection is established, so the connection status between the devices can be known by the transition of the graphs.

In Scenario 1, Device 4 autonomously established connections with Devices 1–3 of the peripheral devices, and we confirmed that sensor data sharing started. According to the results shown in the figure, the connection with Device 2 was established first, after which the connections with the Devices 1 and 3 were established. This is due to the fact that the first device connection to Device 4 was only established via P2P, whereas the devices connections after that needed to switch to a Wi-Fi connection after the P2P connection. Additionally, the reason for the difference in the data acquisition start time between Devices 1 and 3 is that Device 3 failed to establish a P2P and/or Wi-Fi connection and needed to reconnect. An evaluation of the time required for constructing the topology among devices is carried out separately in the next experiment.

In Scenario 2, we confirmed that Device 4 automatically detected the connection established between Devices 1 and 2 while it was in the state in which it was acquiring sensor data only from Device 1, and had started acquiring sensor data from newly recognized devices. We also confirmed that ICOS can communicate not only in a single tree-type topology but also between multiple tree-type topologies.

In Scenario 3, when Device 3 started moving from the state where Device 4 had acquired sensor data from the other three devices, it detached from the network and the provision of sensor data was interrupted. Thereafter, it reconnected to the network as a CL of Device 1 in the destination room and restarted the provision of sensor data to Device 4. From this result, we confirmed that a device could leave the network due to movement, autonomously reconnect to another device in the destination area, and restart sensor data sharing. In addition, as shown in Figure 9c, the section where the sensor value became 0 because of the dropped message was detected. With this point in mind, we verify the stability of ICOS communication in the next experiment.

In Scenario 4, like Scenario 1, the network was autonomously established between the devices, and we confirmed that Device 4 could obtain sensor data from Device 1, which was set at a position where direct communication was not possible.

### 5.3. Performance Evaluation of Topology Construction Mechanism

#### 5.3.1. Setup

In this experiment, we investigated the time required to construct the topology among stationary devices using Devices 1–4 listed in Table 1. We began by arranging the *n* devices in a grid pattern so that the distance between them was 50 cm or 200 cm. Thereafter, after all the devices transited to the “device discovery” state, Device 4 executed “group creation” in order to start the topology construction. We then measured various aspects of the topology construction time (ALL) until the GO became communicable with all the other CLs, the GO authentication time (GO) from the time Device 4 became a GO until a peripheral device discovered and authenticated it, the P2P connection time (P2P) from the time a peripheral device requested a P2P connection to the GO until connection was completed, and the Wi-Fi connection time (Wi-Fi) required for a CL to disconnect its P2P connection and establish a new Wi-Fi connection. The details of these measurement intervals are shown in Figure 4.

#### 5.3.2. Results

**Figure 10:**
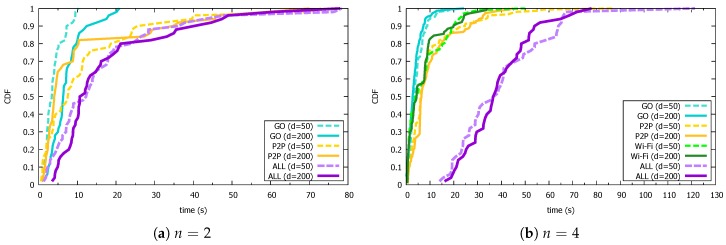
Topology construction results.

Figure 10 shows the results of experiments conducted 50 times. Figure 10a shows the result when n=2, so the Wi-Fi connection time is not measured. In this case, each sample number was 50. In addition, Figure 10a,b shows the result when n=4, and the sample numbers were 50 for ALL, 150 for P2P because three CLs attempted to connect during each trial, and 103 at d=50 and 104 at d=200 for Wi-Fi because two CLs attempted to connect during each trial but managed to reconnect several times.

As can be seen from the figure, in a small environment such as that used in these experiments, ad hoc D2D networks were successfully constructed with no major differences resulting from variations in the distance and the number of devices. More specifically, 90% of all trials completed the connection with a GO authentication time of 10 s, a P2P connection time of 30 s, and a Wi-Fi connection time of 20 s or less. That is, after a certain device started searching for peripheral devices, it was able to complete its connection to the network within 40 s. The relatively large variance seen in the P2P connection times P2P is thought to be because the success rate of negotiations between devices in Wi-Fi Direct is low. In this implementation, when a certain period of time has elapsed since a device attempted P2P connection, it determines that the P2P connection attempt has failed and retries. This appears in the feature that shows P2P connection time in n=2 gradually accumulates stepwise. Therefore, immediately detecting P2P connection failure provides an effective way to shorten the entire topology construction time, as well as improve the stability of Wi-Fi Direct itself.

### 5.4. Performance Evaluation of Multi-Hop Communication Mechanism

#### 5.4.1. Setup

**Figure 11:**
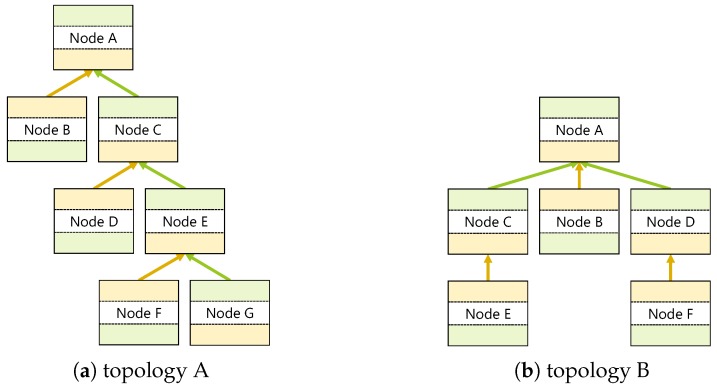
Experiment topologies.

This experiment was conducted to confirm whether multi-hop communication could be performed without delay between arbitrary devices on our ad hoc ICOS network. Figure 11 shows the topology used in this experiment. Among them, Topology A in Figure 11a is a topology with depth priority that was primarily selected to verify whether vertical message exchange in the topology is possible. In contrast, Topology B, which is seen in Figure 11b, is a breadth-first topology that was primarily designed for verifying the possibility of message exchanges in the horizontal direction. Devices 1–7 were used in Table 1 in Topology A and Devices 1–6 were used in Topology B.

In this experiment, devices were spaced 30 cm apart and placed so as to correspond to the built topology. No device received prior information on the other devices or the topology, and only the selection of the GO that became the root node and the target GO were performed manually in order to construct the desired topologies. All devices were kept stationary during the experiment.

The experimental procedure is as follows.

1. Select an arbitrary device as GO and build a new group.
2. Attempt to connect to the GO via Wi-Fi Direct from a peripheral device.
3. Repeat Step (2) for devices that have become new GOs.
4. Repeat Step (3) to build the desired topology.
5. Synchronize the routing tables of the devices.
6. Exchange messages among all devices and measure the success rates and delays.

In the next section, in which we will describe our experimental results, the device alphabet in Figure 11 corresponds with the device numbers given in Table 1. In addition, in this experiment, the regular message exchange function in step (5) in Section 4.1 was invalidated and the routing tables were synchronized between the devices via manual message exchanges. This is to reduce contention between devices and evaluate the influence of the number of hops, and for evaluation purpose, messages were sent one at a time. Note that the experiment results in Section 5.2 show that it is possible to build the topology and exchange messages even if each device freely sends messages.

#### 5.4.2. Results

Table 2 shows the results of D2D communication in Step (6) of the previous section. In this experiment, the average round trip time (RTT) taken and the number of times communication failed were measured while exchanging messages 20 times by the user datagram protocol (UDP) between each pair of devices. From this table, it can be seen that the result varies depending on the device performance or the relationship between devices.

Specifically, when compared with a case in which the transmitting and receiving devices are reversed, even in the same route, such as Node C⇔Node D and Node E⇔Node F in Topology A and Node A⇔Node B in Topology B, we found that the RTT differs greatly. This occurred in GO PB⇔CLP, and similar results were obtained in verifications conducted using different devices. Thus, it is conceivable that processing performance will vary depending on the Wi-Fi Direct role and/or the interface to be used.

Additionally, when examining communications between directly connected devices, we found that CL↔CL communication became slower than GO↔CL communication. In particular, CL↔CL communication in Topology B failed to transmit and receive six times in total. In [30], it was suggested that the packet loss rate increases as the number of groups increases. Therefore, as part of our future work, it will be necessary to verify the influence of CL numbers on communication stability in detail by increasing the number of devices.

**Figure 12:**
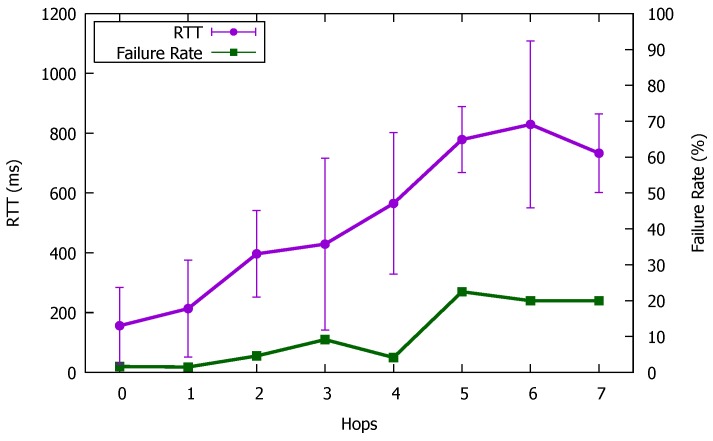
Results for each number of hops.

Figure 12 shows RTT and its standard deviation, and communication failure rates, summarized for the number of hops in each topology. As described above, even when communications use the same number of hops, there is a large variation in the RTTs, but a tendency to roughly increase by 100 ms per hop can be seen. Additionally, we can see that the communication failure rate rose markedly as the number of hops increased.

### 5.5. Discussion

Experiments in Section 5.2 confirmed that multi-hop D2D communication environment can be constructed simply by installing ICOS onto general Android devices. Since ICOS only uses the standard functions of Wi-Fi and Wi-Fi Direct, there is no need to root devices and modify their OSs. Moreover, since it was unnecessary to give peripheral device information required for topology construction and message forwarding in advance, we were able to dynamically construct an ad hoc D2D communication environment by autonomous group management using each GO and construct routing tables based on the relationships between devices. In addition, we confirmed that not only was communication possible within a single ICOS network but also communication between devices in different networks could be performed, even when several ICOS networks were connected by WLAN or when a network is reconstructed as due to device movements. Based on the above, we can conclude that a multi-hop D2D communication platform that satisfies all the requirements described in Section 2.3 has been achieved.

In the experiment of Section 5.3, we confirmed that the devices complete participation to ICOS network in almost 40 s. In addition, in the experiment of Section 5.4, it was confirmed that the communication delay increased about 0.1 s per hop, and the packet loss rate might exceed 20% depending on the situation. From these results, it can be said that ICOS is unsuitable for use in high-mobility environments such as StreetPass communication. However, ICOS can be a useful communication platform for use in disaster environments where communication infrastructures become unstable and the necessity of D2D communication increases. Since ICOS constructs a private ad hoc network instantaneously with unknown peripheral devices and is capable of unicast communication with arbitrary devices, it is possible to construct a topology in various locations such as evacuation shelters, thereby realizing flexible information sharing such as disseminating disaster-related information and exchanging messages among specific users by unicast. In addition, ICOS can be applied to local advertising systems that have limited publication ranges based on their physical geographical area, such as a system that permits users to participate in a local D2D network by entering a specific location such as a sightseeing spot or a restaurant, and receive services such as information sharing and advertisement deliveries that are limited to within the network. It is believed that the application of our proposed network to these systems will further promote data distribution among users and contribute to improvements in the quality and convenience of services.

The simplicity of our algorithm and the ease of setup are the major features of our platform. In other words, unlike related research, it is not necessary to synchronize with peripheral devices or to share information in advance when constructing the topology, and only a slight delay occurs at the time of message transfer. As a result, it can be expected that application to wider areas than existing platform and combination with other related techniques, such as selection method of GO, can be carried out easily. In addition, as for the instability of the communication, using P2P-side NIC and Wi-Fi-side NIC together is considered as one of the causes. Since these are logical interfaces that use the same hardware, switching them is likely to cause delay and packet loss. This is a fundamental problem of the Wi-Fi Direct protocol; therefore, it is necessary to modify the OS in order to solve the problem. As a solution at the application layer, more elaborate implementation such as introduction of a retransmission function can be considered.

Finally, we will list some considerations found through the implementation and the above-mentioned experiments.

**GO power consumption**: When a device acts as a GO, it functions as an AP and waits for connections from peripheral devices. Thus, it was confirmed an apparent increase in power consumption results from maintaining this state. In the use cases presumed in this research, since information is shared by using each user’s smartphone, this burden should not be restricted to the resources of a specific device. Therefore, it will be necessary to introduce a mechanism that provides users some incentive to allow their devices to become GOs, as well as a mechanism that optimizes network topology based on, for example, the geographical location of each device, in order to ensure that it requires only the smallest number of GOs.

**Authentication screen when connecting by Wi-Fi Direct**: According to the Wi-Fi Direct specification, when attempting a P2P connection to GO as a CL, an authentication screen is displayed when there is no previous connection history between the devices. Although this necessarily requires interaction with the device owner when joining the D2D network, the proposed platform cannot avoid this interaction because it requires one or more RNs that have a P2P connection with the GO. Accordingly, it will be necessary to design and develop an application based on the premise of this interaction.

## 6. Conclusions

In our proposed D2D communication platform ICOS, in which the sole requirement is the installation of a dedicated application to each participating device, it is possible to construct a private ad hoc D2D network and share information among unknown neighboring devices. Since preliminary knowledge of, for example, neighboring devices is unnecessary for constructing an ICOS network, an ad hoc network can be constructed instantaneously. In addition, efficient multi-hop communication enables communication between arbitrary devices. Furthermore, we confirmed the above characteristics by implementing the proposed platform using off-the-shelf smartphones and evaluated the network functionality in terms of device performance, communication delays, and stability based on hop number.

As future work, we will pursue a topology construction optimization mechanism by considering mechanisms for selecting RNs that reflect the mobility of each device, its physical location, remaining resource amounts, etc., as well as an appropriate P, W, and B selection mechanism for CLs, and a suitable selection mechanism for the target GO of new participating devices.

## Figures and Tables

**Table 1:** Android devices used.

No.	Product Name	Device Name	Version
1	ZenFone3	ZenFone3-01	7.0
2	ZenFone3	ZenFone3-02	7.0
3	ZenFone3	ZenFone3-03	7.0
4	Xperia Z3 Compact	XperiaZ3C	6.0.1
5	GALAXY S4	SC-04E-01	5.0.1
6	GALAXY S4	SC-04E-02	5.0.1
7	Ascend G620S	HUAWEI-01	4.4.4

**Table:** (**a**) Topology A

src. \ dst.	A	B	C	D	E	F	G
**A**	-/-	114/0	126/0	260/1	439/1	661/1	758/5
**B**	55/0	-/-	146/1	388/1	593/5	758/3	925/4
**C**	130/0	182/1	-/-	187/0	225/0	400/2	535/1
**D**	441/4	356/3	33/0	-/-	74/0	309/0	511/2
**E**	575/0	589/4	224/1	208/0	-/-	127/0	210/0
**F**	873/0	804/6	331/3	357/0	90/0	-/-	148/0
**G**	712/3	918/11	503/0	461/0	165/0	112/0	-/-

**Table:** (**b**) Topology B

src. \ dst.	A	B	C	D	E	F
**A**	-/-	127/0	308/0	276/0	298/0	565/0
**B**	28/0	-/-	302/2	241/0	338/0	303/0
**C**	267/0	325/0	-/-	345/2	51/0	550/0
**D**	203/2	250/2	355/0	-/-	345/2	157/0
**E**	317/0	426/2	58/0	424/1	-/-	761/0
**F**	441/1	458/2	507/0	87/0	753/3	-/-
